# Support Screening to Shape Propane Dehydrogenation SnPt-Based Catalysts

**DOI:** 10.1021/acs.iecr.3c04089

**Published:** 2024-09-12

**Authors:** Giovanni Festa, Ana Serrano-Lotina, Eugenio Meloni, Raquel Portela, Concetta Ruocco, Marco Martino, Vincenzo Palma

**Affiliations:** † Department of Industrial Engineering, University of Salerno, Via Giovanni Paolo II 132, 84084 Fisciano, Salerno, Italy; ‡ Instituto de Catalisis y Petroleoquimica (ICP), CSIC, C/ Marie Curie 2. 28049 Madrid, Spain

## Abstract

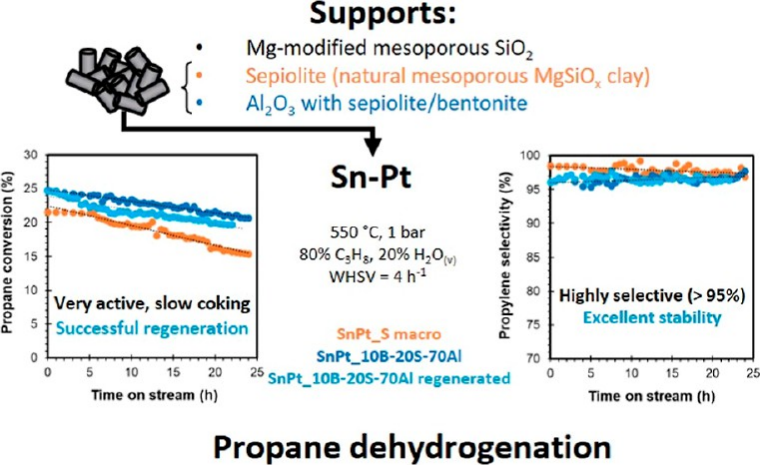

Propane dehydrogenation reaction (PDH) is an extremely attractive way to produce propylene; however, the catalysts often lead to byproduct formation and suffer from deactivation. This research focuses on the development of efficient Pt/Sn-based shaped catalysts by utilizing Mg-modified mesoporous silica, sepiolite (natural SiMgO_*x*_ mesoporous clay), and sepiolite/bentonite/alumina as supports with the aim of achieving superior stability and selectivity for industrial propylene production by PDH. The catalysts were prepared by sequential impregnation of the supports with the corresponding solutions of tin chloride and platinum chloride, by obtaining a nominal loading of 0.7 wt % of Sn and 0.5 wt % of Pt. A range of analytical techniques were used to characterize the catalysts, including X-ray diffraction, nitrogen physisorption isotherms, Hg intrusion porosimetry, thermogravimetric analyses, transmission electron microscopy, Raman spectroscopy, and X-ray photoelectron spectroscopy. The basicity of the catalysts was assessed using carbon dioxide temperature-programmed desorption (CO_2_-TPD). The results confirm that the support material plays a critical role in catalyst performance; in particular, the presence of weak basic sites, due to magnesium addition, improved selectivity to propylene and reduced coke formation. Catalytic pellets of Sn–Pt supported on macroporous sepiolite or sepiolite and bentonite-modified mesoporous alumina performed comparably with propane conversion very close to thermodynamic equilibrium and selectivity to propylene above 95%. The latter support led to improved stability and was regenerated at milder temperatures, making it suitable for industrial applications.

## 1 Introduction

Propylene is a key element of the chemical industry and is the feedstock to produce polypropylene, which is one of the most widely used plastics. Propylene is conventionally obtained as a byproduct by fluid catalytic cracking or by naphtha and light diesel steam cracking processes; however, the growing global demand led to an increased interest toward the study of specific propylene production processes. From this prospective, active catalysts for propane dehydrogenation (PDH) can produce propylene with 99% selectivity.^1^ However, due to its endothermicity, PDH requires relatively high operating temperatures to obtain high propylene yields, which in turn favor thermal cracking reactions to coke and light alkanes that lead to a decrease in selectivity and an increase in catalyst deactivation.^2^ While chromium- or platinum-based catalysts have been widely studied for the PDH reaction,^3^ bimetallic catalysts can reduce the coke formation rate, and thus the frequency of the catalyst regenerative cycles, and optimize the operating conditions. Many studies demonstrated that the addition of Sn to Pt-based catalysts suppresses some side reactions, thus increasing both the selectivity to propylene and the catalyst stability.^4^ The support has a crucial role for the catalytic activity. γ-alumina is the most widely used support for PDH catalysts, due to its advantageous textural properties and low cost,^5,6^ but the Lewis acid sites on its surface are directly related to coke formation, strongly affecting the catalytic performance, and therefore, it is necessary to adjust the surface acid sites to achieve superior stability.^7^ An alternative suitable support for PDH is silica, due to its excellent thermostability, availability, relatively high surface area, and inertness.^8,9^ Sricharoen et al. carried out a comparative study of SiO_2_- and Al_2_O_3_-supported catalysts, observing coke formation for both, due to the typical acidity of both supports, but by different mechanisms that implicate different catalytic performance.^10^ The SiO_2_-supported catalyst favors catalytic cracking and therefore most of the coke deposits at the support; instead, coke formation on catalysts supported on Al_2_O_3_ involves hydrogenolysis and hydrogenation reactions that lead the coke to deposit onto both the active sites and the support. As a result, the use of SiO_2_ enhances the catalytic performance. Particularly interesting is mesoporous silica, since its highly organized porosity and high surface area may be useful features in PDH.^11^ In a recent study, Wang et al. have demonstrated a correlation between the pore size distribution of siliceous supports and the catalyst performance in PDH.^12^ Moreover, supports with 3D interconnected mesopores overcome the mass transfer disturbance and enhance the catalytic performance. Anyway, support acidity remains a key parameter to be controlled for the development of a more active, selective, and stable catalyst tuning, and three strategies have been identified: (i) chemical promoters, (ii) doped supports, and (iii) more basic supports. Chemical promoters include alkali and alkaline—earth metals as calcium^13^ or potassium,^14^ which are able to provide excess mobile electrons to the Pt-based catalyst and stabilize the active sites, thus reducing the coke formation rate. Doping of the support with rare earths such as yttrium,^15^ lanthanum,^16^ and cerium^17^ as well as with magnesium^18^ reduces the support acidity and thus coke formation during reaction. Among the others, it is worth noting that the basic MgO support is a good electron donor, which can weaken the surface acidity of supports and modulate the electronic structure of active metal phases.^19^ In particular, nonacidic materials, especially magnesium aluminum oxides [Mg(Al)O], have received considerable attention as supports for PDH catalysts owing to their moderate acid characteristics, high thermal stability, and high metal dispersion.^20^ Moreover, the Mg(Al)O support could greatly suppress coke deposition, minimize the adsorption of alkenes, as well as modify the properties of Pt particles and provide the strong metal-support interaction.^21^ In fact, an appropriate amount of magnesium in Pt–Sn-based catalysts can not only decrease the support acidity, but also improve the Sn interaction with both Pt and support, leading to a greater proportion of Sn in its oxidized states, thus decreasing aggregates of metal particles and enhancing catalytic performance.^22^ For the third strategy, materials containing basic oxides in the chemical structure allow us to achieve very interesting, modified supports. Sepiolite, which is a fibrous clay mineral composed of blocks of magnesium oxide/hydroxide octahedral sheets inserting between two layers of tetrahedral silica [formula Mg_4_Si_6_O_15_(OH)_2_·6H_2_O], may be a promising material. The constituent blocks have discontinuities in the octahedral silica sheets that lead to the construction of structural channels and tunnels.^23^ This peculiar structure gives the sepiolite fibers a high specific surface area, high porosity, and a large quantity of surface hydroxyl groups.^24^

As mentioned before, the PDH reaction pathway is complex and involves many side reactions, including cracking, hydrogenolysis, and polymerization, which inevitably lead to coke formation, which is easily deposited both on the Pt active sites and the support.^24^ Many efforts have been made to understand the coke nature and formation mechanism and to enhance the catalyst stability and the propylene yield by developing new high-performance catalysts. Larsson et al.^25^ reported that coke formation depends mainly on the operating temperature and that only a small part of the formed coke is responsible for Pt/Al_2_O_3_ and Pt–Sn/Al_2_O_3_ catalyst deactivation. Furthermore, in transient experiments using the same catalyst, they identified reversible and irreversible coke.^26^ Li et al. identified two types of coke over a Pt–Sn/Al_2_O_3_ catalyst: one with aliphatic hydrocarbons, which tends to deposit on the metal, and one with aromatic hydrocarbons, which tends to deposit on the support.^5^ Redekop et al. reported that the coke on a Pt/Mg(Al)O_*x*_ catalyst was composed of graphene sheets.^6^ They also found that the graphene sheets formed on the small Pt particles (1.5–2.0 nm) shifted continuously on the support. On the contrary, the graphene formed on the larger Pt particles (5–10 nm) tended to form encapsulating graphitic layers. In addition to coking directly caused by active sites, Lewis or Bronsted acidic sites also catalyze the coking process like a catalyzed aromatic hydrocarbon process and follow an acid-catalyzed carbocation mechanism.^27^

With the aim to design highly active and stable catalysts for industrial propylene production, different Pt/Sn-based catalysts were prepared using Mg-modified mesoporous silica, sepiolite, and a sepiolite/bentonite/alumina as supports to take advantage of the synergy among magnesium, silicon, and aluminum oxides. The catalysts have been tested with optimized steam content in the feed, taking advantage of the fact that steam can act as heat carrier toward the catalytic system, as well as suppress coke deposition;^28^ moreover, steam addition results in a dilution of the system, thermodynamically promoting propane conversion. On the other hand, the presence of steam could generate a reforming reaction, reducing selectivity toward propylene.

## 2 Experimental

### 2.1 Catalysts Preparation

Six different supports (Table S1) were used to support the bimetallic active phase: three were based on pearls of mesoporous silica (mSiO_2_) with dimensions of 1–2.3 mm provided by ROTH, as received or modified with 3 or 7% Mg (3Mg_SiO_2_ and 7Mg_mSiO_2_), and the other three were clay-based pellets composed of sepiolite (S-500 and S-Macro, both calcined at 500 °C) and sepiolite-modified γ-alumina (10B20S70A, containing 10 wt % bentonite, 20 wt % sepiolite, 70 wt % alumina, calcined at 550 °C) provided by CSIC.^29^ The clay-based pellet supports were manufactured by extrusion of Pansil 100 and Pangel S9 natural sepiolites (60 and >85% purity, respectively) and Pangel M280 bentonite, all from Tolsa SA, as well as high purity Pural SB1 boehmite from Sasol. For extra macroporosity generation, 40% activated carbon was included into the dough of S-Macro and then removed during calcination.^30^ The extrudates were crushed and sieved between 1 and 2.5 mm for further use.

The catalysts (Table S2) were prepared by sequential wet impregnation of the as received supports with an ethanol solution of tin chloride and an aqueous solution of platinum chloride, with a nominal loading of 0.7 wt % of Sn and 0.5 wt % of Pt, respectively. Platinum(IV) tetrachloride 99% and ethanol were supplied by Carlo Erba, tin(II) chloride 98% was supplied by Sigma-Aldrich, and distilled water was supplied by Best Chemical. After each impregnation, the samples were dried for 24 h at 80 °C, dried for 2 h at 120 °C, and finally calcined for 3 h at 600 °C.

### 2.2 Catalytic Activity Tests

The catalytic activity tests were performed under atmospheric pressure at a weight hourly space velocity (WHSV, calculated as the ratio between the total mass flow rate and the mass of catalyst) of 4 h^–1^ in a stainless-steel tubular reactor with an internal diameter of 1.4 cm and a length of 50 cm loaded with 13.5 g of catalyst. The reactor outlet stream was dried through a refrigerator Julabo F12 and sent to a gas chromatograph Agilent Technologies 7820A, equipped with an FID (flame ionization) and a TCD (thermo conductivity) detector, for evaluating the molar fraction of propane, propylene, hydrogen, CO, CO_2_, methane, ethane, 1-butene, iso-butane, and *n*-butane.

The catalysts were always activated in situ at 600 °C under a flow rate of 100 N mL min^–1^ g_cat_^–1^ by a three-step procedure consisting of the reduction in 5 vol % H_2_/N_2_ for 1 h, followed by oxidation in 5 vol % O_2_/N_2_ for 1 h, and subsequent reduction in pure hydrogen for 1 h. A preliminary screening of the catalyst’s activity was performed in the temperature range of 490–600 °C with a reacting mixture composed of 20 vol % H_2_O and 80 vol % propane. The stability and regenerability of the best performing catalysts in terms of propylene yield were evaluated by a procedure consisting of PDH reaction at 550 °C for 24 h, followed by cooling down to 500 °C in N_2_ flow (≈20 min) with subsequent gradual increase in O_2_ concentration from 2% to 20% (≈3.5 h) to burn the coke and final reduction in 5 vol % H_2_ in N_2_ flow at 600 °C (20 min for heating and then 1 h dwell time). The regenerated catalyst was then submitted to PDH reaction conditions for 24 h.

The catalytic activity was evaluated in terms of propane conversion , selectivity to propylene (*S*_C_3_H_6__), selectivity to coke (*S*_coke_, from carbon balance), and propylene yield (), calculated with the following equations
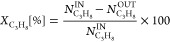
1
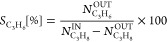
2
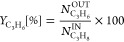
3
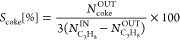
4In which *N*_*j*_^*i*^ are the molar flow rates of the components. From the carbon balance, considering all of the carbon containing products at the inlet and outlet of the catalytic system, we obtained *N*_coke_^OUT^ for the calculation of the selectivity to coke. The carbon balance can be considered an accurate method for coke estimation since no other carbon-containing products have been detected during the analysis (Figure S3 shows an example of peak identification during GC analysis of the PDH test). In any case, controlled coke oxidation tests have been performed at 500 °C with the spent catalysts (see an example in Figure S4) to compare the calculated coke amounts with those determined experimentally and, thus, validate the accuracy of the carbon balance. The coke deposits determined as the sum of the areas under the mass spectrometer (MS) peaks of CO and CO_2_ were very close to the values calculated with the carbon balance, confirming the feasibility of the mathematical approach.

The deactivation behavior has been evaluated by means of a deactivation constant, calculated as follows^31,32^
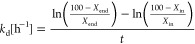
5

In which *X*_in_ is the initial propane conversion and *X*_end_ is the propane conversion at the end of the stability test.

### 2.3 Catalysts Characterization

The supports and the catalysts, fresh (f), after activation by reduction in H_2_ (a), and spent after 24 h on stream (s), were characterized by means of a series of analytical techniques. Energy-dispersive X-ray fluorescence was performed by an ARL QUANT’X ED-XRF spectrometer (Thermo Fisher Scientific), in order to check the effective metal loading. X-ray diffraction (XRD) was used to evaluate the crystalline structure in a PANalytical X’Pert Pro diffractometer using Ni-filtered Cu Kα radiation with λ = 1.5406 nm. Nitrogen adsorption–desorption isotherms measured at 77 K (Quantachrome Instruments, mod. Nova 1200e) were used for the calculation of the specific surface area through the Brunauer, Emmet, and Teller equation and for the evaluation of the pore size distribution through the BJK method. Structural characterization by Raman spectroscopy was performed by using an inVia Raman Microscope (Renishaw) equipped with a 514 nm Ar ion laser, operating at 25 mW. Field emission scanning electron microscopy (FE-SEM) was performed with a FEI Nova NanoSEM 230 microscope; the samples were gold-sputtered for 25 s in a Leika EM ACE200 coating system at 30 mA and 0.5 bar Ar. Transmission electron microscopy (TEM) characterization was used to evaluate the particle size and shape and was carried out in a JEOL Model JEM-1200 EXII transmission electron microscope equipped with an emission source of electron at 200 kV. Samples were ultrasonically dispersed in ethanol, and a few drops were placed on a holey-carbon-coated copper grid, allowing the solvent to evaporate in air before TEM observation. Thermogravimetric analyses (TGA) were performed by a Q600 connected to a quadrupole-mass spectrometer (MS) detector (Discovery MS, TA Instruments). TGA was conducted from 25 to 1000 °C with a heating rate of 10 °C min^–1^ and an air flow rate 100 N mL min^–1^. X-ray photoelectron spectroscopy spectra were recorded using a spectrometer with a UHV system (pressure: 10^–10^–10^–9^ mbar), a PHOIBOS 150 9MCD analyzer, and a Multi-Channeltron detector. The used excitation source was Al K_α_ radiation (*h*υ = 1486.6 eV; 200 W; 12 kV; pass energy = 20 eV). The pellets were analyzed without crushing them to avoid sample modification. Positive charge compensation was performed using adventitious carbon as an internal reference (C 1s: 284.6 eV). Pt 4f overlaps with Al 2p, so this area has been treated carefully, considering the Pt 4f_7/2_–4f_5/2_ splitting: 3.35 eV and the ratio between its areas (4:3). For Sn 3d fitting, we have considered a Sn 3d_5/2_–3d_3/2_ splitting of 8.5 eV and a ratio between the areas of 2:3.

The basicity of the catalysts was studied by carbon dioxide temperature-programmed desorption (CO_2_-TPD) with 5 g of the sample in a tubular reactor with an internal diameter of 14 mm. After aging pretreatment for 5 h under 10 vol % H_2_ in Ar (100 N mL min^–1^ g_cat_^–1^) at 580 °C to simulate the reaction conditions, the samples were cooled down and treated in 10 vol % CO_2_ in Ar at 50 °C for 1 h, followed by purging with Ar. CO_2_-TPD was then performed under Ar flow in the temperature range 25–680 °C (heating rate of 10 °C min^–1^). The reactor outlet was analyzed by means of a MS Pfeiffer OMNISTAR.

## 3 Results

### 3.1 PDH Catalytic Activity

**Figure 1:**
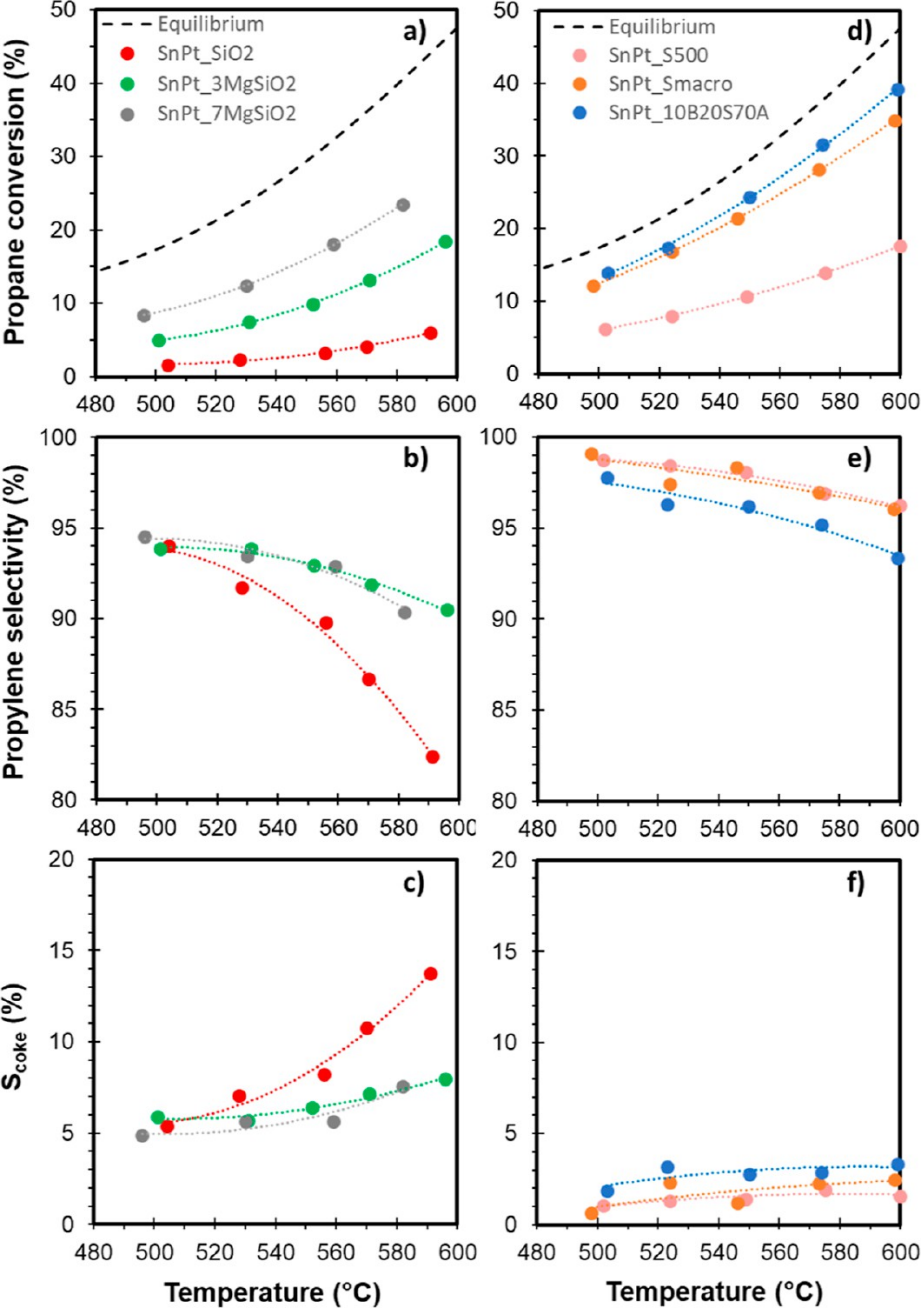
Propane conversion (top), propylene selectivity (middle), and *S*_coke_ (bottom) vs temperature. Comparison among catalysts based on mesoporous silica (SnPt_SiO_2_—red, SnPt_3MgSiO_2_—green, and SnPt_7MgSiO_2_—dark gray, on the left) and on sepiolite (SnPt_S-Macro—red, SnPt_S-500—pink, and SnPt_10B20S70A—blue, on the right). C_3_H_8_/H_2_O = 8/2, 1 bar, WHSV = 4 h^–1^.

Figure 1 shows the catalytic activity between 490 and 600 °C for the mSiO_2_-based SnPt catalyst series on the left, and the catalysts supported on sepiolite (S-500 and S-Macro) and on γ-alumina extruded with sepiolite and bentonite (10B20S70A) on the right in terms of propane conversion and selectivity to propylene and coke.

#### 3.1.1 Magnesium-Modified Mesoporous Silica-Supported Catalysts

Figure 1a evidences that the SnPt_SiO_2_ sample, without Mg, has very low activity, but the addition of Mg progressively enhances the performance. In fact, SnPt_7MgSiO_2_, with the highest Mg loading in the mSiO_2_-based series, converts 23.5% of the propane in the feed at 580 °C, a value, however, still far from the thermodynamic equilibrium. The selectivity to propylene (Figure 1b) and thus the tendency to form coke (Figure 1c) underline the positive influence of magnesium on the catalytic performance. At 500 °C, propylene selectivity is similar in Mg- promoted and unpromoted catalysts (∼95%), but at higher temperature, and thus conversion, it significantly drops in the catalyst without Mg, whereas SnPt_3MgSiO_2_ and SnPt7_MgSiO_2_ catalysts maintain selectivity values over 90% despite their conversion is several times higher. Accordingly, the carbon mass balance indicates the positive influence of magnesium to avoid the formation of compounds with high carbon content, including coke, observed at temperatures higher than 530 °C in the absence of Mg. The results demonstrate that increasing the amount of magnesium in the catalytic support improves conversion and selectivity. Therefore, new catalysts were prepared by using supports in pellet form that contain magnesium in their composition from natural clays.

#### 3.1.2 Sepiolite-Supported Catalysts

SnPt_S-Macro and SnPt_10B20S70A catalysts showed comparable propane conversion, close to the thermodynamic equilibrium, with values at 600 °C of 35 and 39%, respectively, while the activity of SnPt_S-500 was significantly lower, with 18% conversion at 600 °C (Figure 1d). The selectivity to propylene was similar for the three catalysts (93% at 600 °C), although slightly lower for SnPt_10B20S70A (Figure 1e), and consequently, the carbon balance followed an opposite trend (Figure 1f). Compared to the performance of the best catalyst in the mSiO_2_ series (SnPt_7MgSiO_2_), characterized by a slightly lower selectivity and a maximum conversion of only 23.5%, the results obtained with the catalysts based on sepiolite, which naturally contains 23.7 wt % of MgO (14.2 wt % Mg), either combined with alumina or with extra macroporosity, are outstanding.

### 3.2 Stability

**Figure 2:**
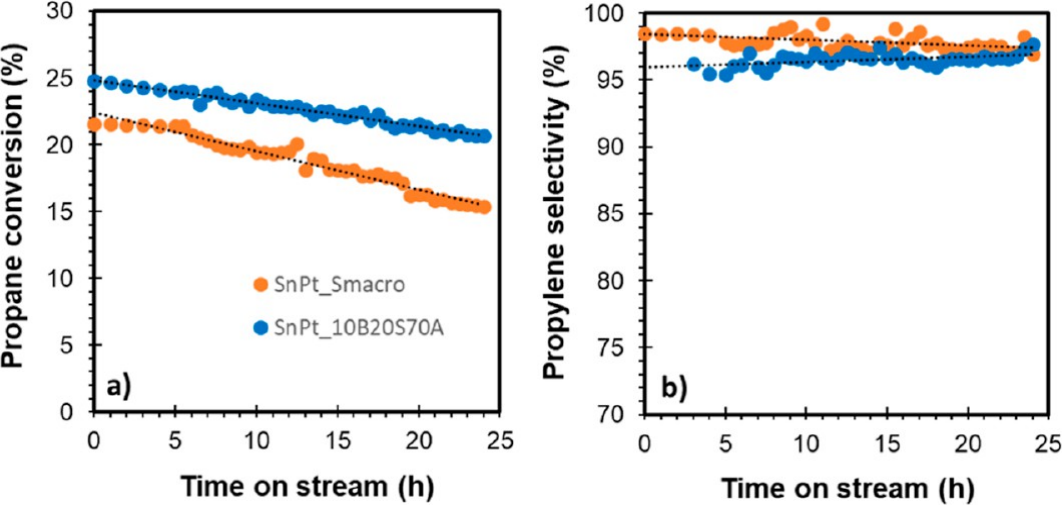
Stability test. Propane conversion (a) and propylene selectivity (b) over time. Comparison between SnPt_S-Macro (orange) and SnPt_10B20S70A catalyst (blue) (550 °C, 1 bar, WHSV = 4 h^–1^).

The most promising catalysts, SnPt_10B20S70A and SnPt_S-Macro, were tested for stability during 24 h of time on stream at 550 °C. In both cases, propane conversion decreased over time; however, the SnPt_S-Macro catalyst deactivated faster than SnPt_10B20S70A (Figure 2a), with a deactivation constant of 0.021 and 0.008 h^–1^, respectively (Table 1).

**Table 1:** Stability Test Results

catalyst	propane conversion (%)		propylene selectivity (%)		*k*_d_ (h^–1^)
	after 1 h	after 24 h	after 1 h	after 24 h	
SnPt_10B20S70A	24.6	21.3	96.1	96.8	0.008
SnPt_S-Macro	23.2	15.44	98.4	96.9	0.021

Propylene selectivity decreased over time over SnPt_S-Macro, while it remained almost constant (a slight increase was even detected) over SnPt_10B20S70A (Figure 2b, Table 1).

### 3.3 Characterization of the Catalysts

The textural properties of the supports and the catalysts are summarized in Tables S1 and S2, respectively, in the Supporting Information. Moreover, in Table S2, the active metal loading is also reported. The magnesium-modified mesoporous silica supports are characterized by a very high value of *S*_BET_, even if a decreasing trend from 546 up to 268 m^2^ g^–1^ is visible with the increase in Mg addition from 0 to 7 wt %. The pore volume measured by mercury intrusion porosimetry of the sepiolite material (S-500) was doubled when it was extruded with activated carbon as pore generation agent (S-macro), and the surface area (measured by N_2_ adsorption/desorption isotherms) decreased from 164 to 141 m^2^ g^–1^. The same trend was observed with the Pt–Sn based catalysts.

**Figure 3:**
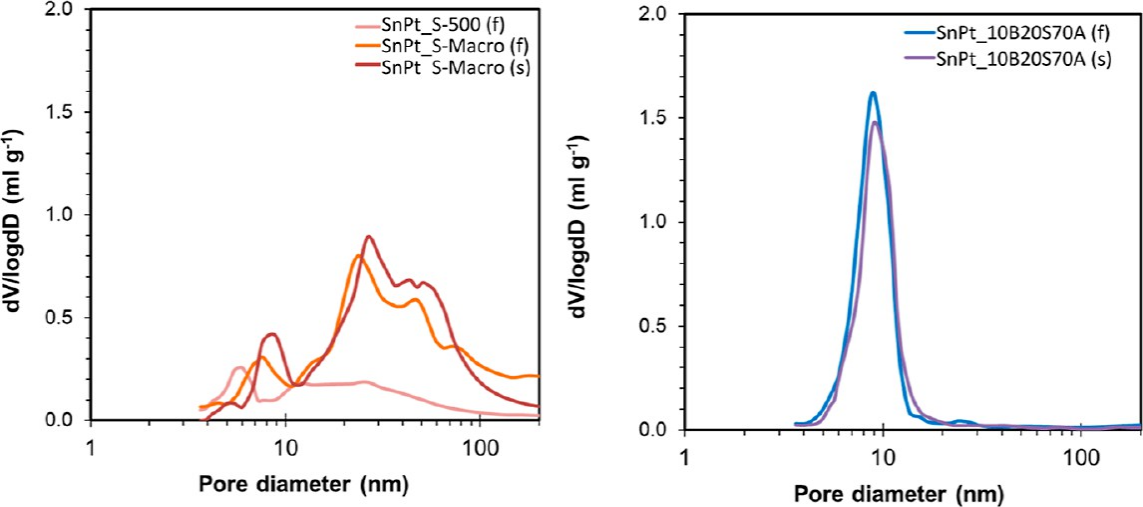
Pore size distribution of the shaped catalysts as obtained by mercury intrusion porosimetry. (left) Sepiolite supports. (right) Alumina-containing support.

Figure 3 compares the pore size distribution of the clay-based fresh (f) catalysts, showing the broad pore size distribution of the sepiolite materials, with meso- and macropores up to 100 nm, and the monomodal distribution centered at 9 nm of the alumina-containing materials. After the stability test, the porosity of the spent (s) catalysts was slightly lower; therefore, the pore area of S-Macro and 10B20S70Al supported samples was reduced to 110 and 147 m^2^ g^–1^, respectively.

**Figure 4:**
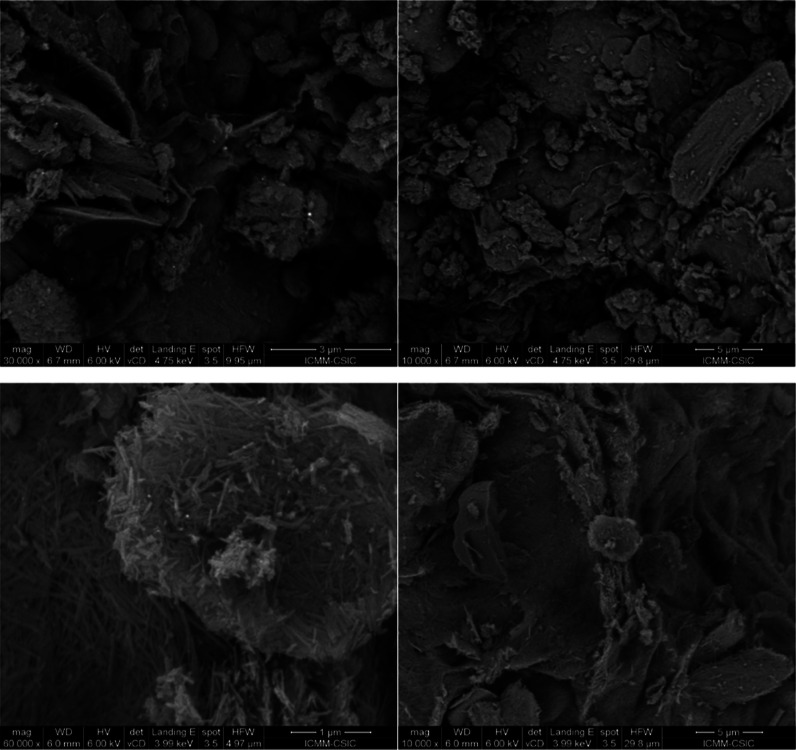
FE-SEM images of SnPt_10B20S70A (top) and PtSn_S-Macro (bottom).

EDS analyses (Figure S1) confirmed the presence of the Pt and Sn active phases in all samples. In the fresh catalysts, Cl traces from the precursors were clearly present but disappeared in the spent samples. Additional EDS analyses indicated that Cl was already removed from the samples after activation (not shown). FE-SEM micrographs in Figure 4 show the different morphology of the most active samples. SnPt_S-Macro shows the typical fibrous shape of sepiolite, while in SnPt_10B20S70A the fibers, in minor proportion, surround the alumina particles.

**Figure 5:**
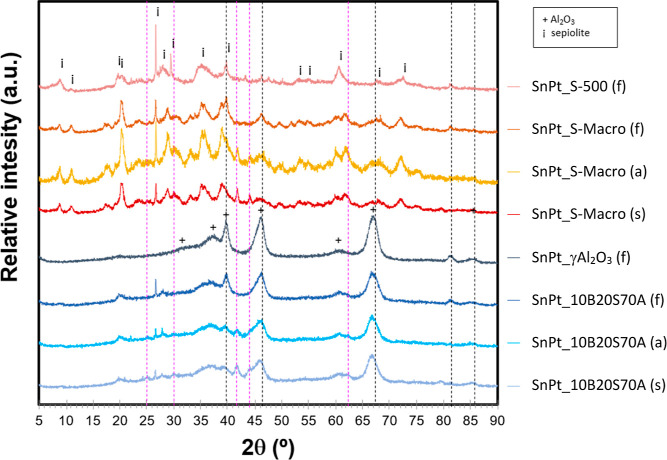
XRD patterns of the PtSn catalyst series on sepiolite-based supports (pure sepiolite and alumina-containing materials, pure alumina included as reference). Suffixes in the samples indicate fresh (f), activated (a), or spent after 24 h operation for the PDH reaction (s). Symbols: alumina (+), anhydrous sepiolite (i), metallic Pt (dashed gray lines), and PtSn alloy (dashed pink lines). Reference XRD patterns of relevant Pt and Sn species are in Figure S2.

The XRD peaks of the different supports are clearly identified in all fresh, activated, and spent samples, as shown in the diffractograms in Figure 5. Diffraction peaks related to Pt or Sn species (Figure S2) were difficult to detect in the samples due to their low content and high dispersion and to signal overlapping. Peaks of Sn^0^ should appear at ca. 30.6° (200), 32° (101) and 44.9° (211) (JPDS # 00-004-0673), but they are not detected in any sample, neither are SnO_2_ diffractions (JPDS # 00-029-1484), which may indicate that Sn is present only as amorphous phases or with very small crystalline domains. Even though the main peaks of metallic Pt at 39.7° (111), 46.3° (200) and 67.4° (220) (JPDS # 00-004-0802) overlap with peaks of alumina and sepiolite, Pt^0^ diffractions can be distinguished in the fresh samples. Their relative intensity is lower in the samples activated by reduction with H_2_ and in samples used as catalysts for PDH reaction, while new peaks appear, which may be attributed to the formation of a PtSn alloy with diffraction peaks located at 2θ values of 41.8° (102), 44.1° (110), 62.5° (202), 30.1° (101), and 25.1° (100) (JPDS # 00-025-0614).^33,34^ PtSn and Pt_3_Sn alloy formation in Pt–Sn-based PDH catalysts has already been reported in the literature, as well as Pt encapsulation by its alloys with Sn.^34,35^ In this study, Pt_3_Sn would be difficult to detect by XRD due to its coincidence with Pt and support diffraction peaks. The formation of alloys with Sn would explain the lower intensity of the Pt^0^ peaks, although the formation of small PtO_*x*_ domains cannot be discarded. Formation of SnO_*x*_ cannot be assessed by XRD due to signal overlapping with the PtSn alloy of the peak at 2θ = 29.39°.

**Figure 6:**
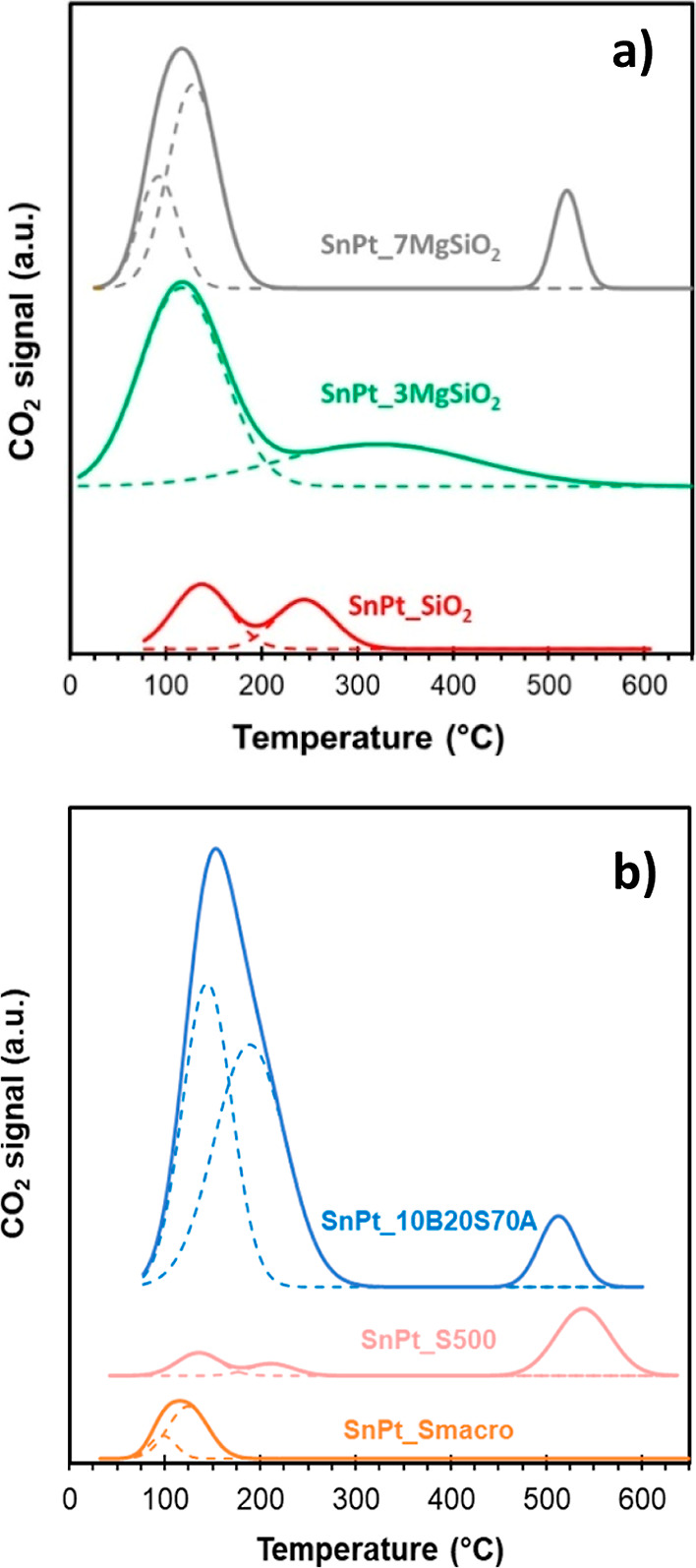
CO_2_-TPD profile for (a) *m*-SiO_2_-based and (b) sepiolite-based catalysts.

Figure 6a shows the CO_2_-TPD profiles over the *m*-SiO_2_ based catalysts. Generally, three main zones are distinguished in these profiles: (i) the CO_2_ desorption peaks at lower temperatures (100–250 °C) are associated with weak Brønsted basic sites such as surface −OH groups; (ii) the CO_2_ desorption peaks at intermediate temperatures (250–400 °C) are associated with medium-strength Lewis basic sites, and (iii) the higher-temperature CO_2_ desorption peaks (400–600 °C) are associated with low-coordination oxygen anion adsorption and act as strong basic sites.^36^ For the SnPt_SiO_2_ sample, a very small double peak was observed between 100 and 300 °C, demonstrating the medium-weak CO_2_ desorption, consistent with previous observations^37^ In contrast, the addition of Mg oxide to the support enhanced CO_2_ adsorption, which is related with a higher number of basic sites, both for the SnPt_3MgSiO_2_ and the SnPt_7MgSiO_2_ samples.^38^ As reported in Table S3, the amount of desorbed CO_2_, calculated from the integration area of desorption profiles and normalized by the catalytic mass, increased in the order SnPt_SiO_2_ ≪ SnPt_3MgSiO_2_ < SnPt_7MgSiO_2_. Thus, mSiO_2_-supported catalysts are mostly characterized by weak basic hydroxyl groups, and medium basic sites, such as Mg^2+^ and the O^2–^ pair, are detected over 500 °C only in the sample with 7% Mg.

The CO_2_-TPD profile of the sepiolite-based catalysts can be observed in Figure 6b. The SnPt_10B20S70A catalyst has the main desorption peaks in the weak Brønsted basic sites temperature range and a small peak located close to 500 °C; the CO_2_-TPD profile of SnPt_S-500 catalyst has three peaks, two small peaks in the weak Brønsted basic sites region and the main peak located over 500 °C; finally, the CO_2_-TPD profile SnPt_S-Macro shows peaks in the weak Brønsted basic sites only, with a total CO_2_ uptake of 3.73 μmol·g_catalyst_^–1^. This value is slightly lower than the 4.81 μmol·g_catalyst_^–1^ of SnPt_S-500, like the values of the Mg-containing catalysts of the *m*-SiO_2_ series. Conversely, the 10B20S70A alumina-rich support provided ca. a 10 times higher number of basic sites than the rest of Mg-containing supports, with a total CO_2_ uptake of 31.7 μmol·g_catalyst_^–1^. Moreover, their strength is lower, as the fraction of weak acid sites is higher, desorbing CO_2_ at lower temperature than in the SnPt_7MgSiO_2_ and the SnPt_S-500 samples, which is expected to be beneficial, as strong basic sites may hinder propane adsorption during the dehydrogenation reaction.^39^

**Figure 7:**
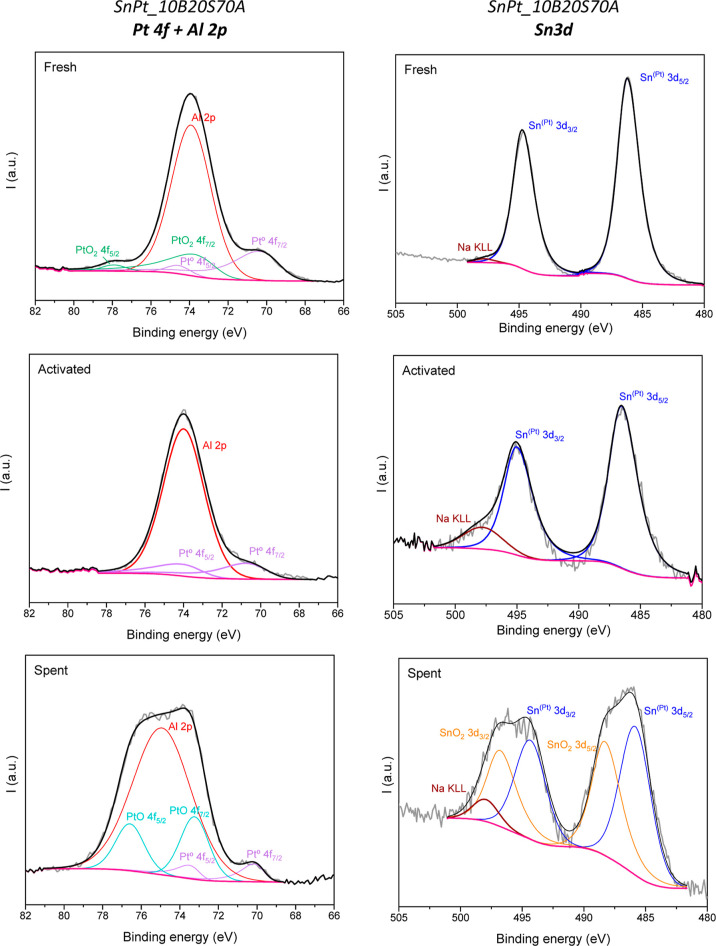
XPS results for SnPt_10B20S70A catalyst fresh (top), activated by reduction (medium) and spent after 24 h on stream for PDH reaction (bottom). Left: Pt 4f + Al 2p. Right: Sn 3d. Color code: raw spectra: gray, background: magenta, sum of fitted spectra: black, fitted spectra are Al 2p: red, PtO_2_ 4f: green, Pt^0^ 4f: violet, PtO 4f: dark cyan, Sn^(Pt)^ 3d: blue, SnO_2_ 3d: orange, Na KLL: brown.

**Figure 8:**
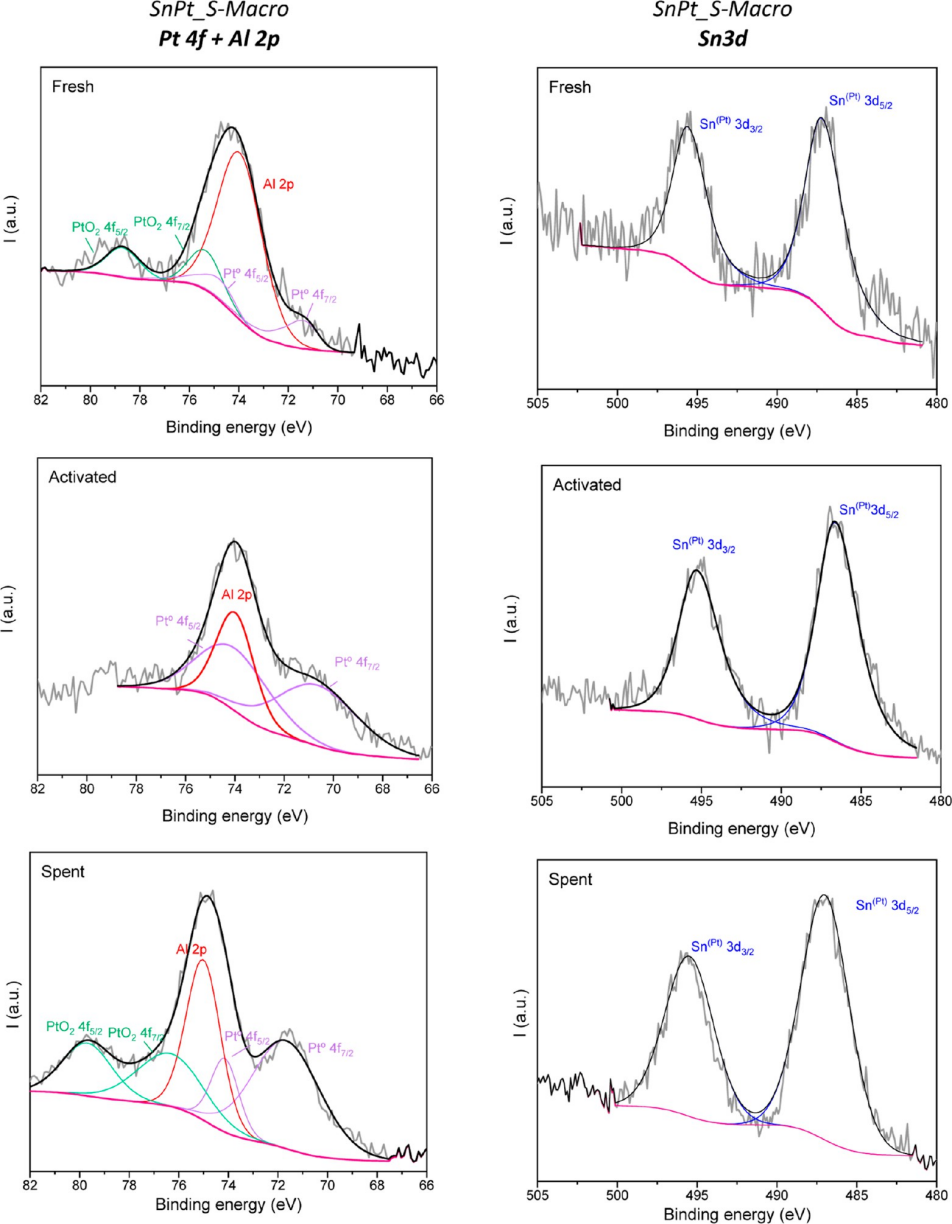
XPS results for SnPt_S-Macro catalyst fresh (top), activated by reduction (medium), and spent after 24 h on stream for PDH reaction (bottom). Left: Pt 4f + Al 2p. Right: Sn 3d color code: raw spectra: gray, background: magenta, sum of fitted spectra: black, fitted spectra are Al 2p: red, PtO 4f: green, Pt^0^ 4f: violet, Sn^(Pt)^ 3d: blue.

Figures 7 and 8 summarize the XPS results for SnPt_10B20S70A and SnPt_S-Macro, respectively, and Table 2 summarizes the fitting details.

**Table 2:** XPS Fitting Details of Fresh, Activated, and Spent SnPt_10B20S70A and SnPt_S-Macro Samples

SnPt_10B20S70A (f)							
	Al 2p	Pt^0^ (4f_7/2_)	Pt^0^ (4f_5/2_)	PtO_2_ (4f_7/2_)	PtO_2_ (4f_5/2_)	SnO_*x*_ (3d_5/2_)	SnO_*x*_ (3d_3/2_)
Bkg	Shirley	Shirley	Shirley	Shirley	Shirley	Shirley	Shirley
peak shape	GL (30)	LA (1.2,85,70)	LA (1.2,85,70)	GL (30)	GL (30)	LA (3,2,0)	LA (3,2,0)
area	8277	2240	1680	269	202	23,989	15,993
fwhm	2.4	2.4	2.4	1.4	1.4	1.9	1.8
position	73.9	69.7	73.0	74.6	77.9	486.2	494.7

SnPt_10B20S70A (a)					
	Al 2p	Pt^0^ (4f_7/2_)	Pt^0^ (4f_5/2_)	SnO_*x*_ (3d_5/2_)	SnO_*x*_ (3d_3/2_)
Bkg	Shirley	Shirley	Shirley	Shirley	Shirley
peak shape	GL (30)	LA (1.2,85,70)	LA (1.2,85,70)	LA (3,2,0)	LA (3,2,0)
area	7225	1065	798	8530	5687
fwhm	2.5	2.4	2.4	2.4	2.4
position	74.0	70.0	73.4	486.5	495.0

SnPt_10B20S70A (s)									
	Al 2p	Pt^0^ (4f_7/2_)	Pt^0^ (4f_5/2_)	PtO (4f_7/2_)	PtO (4f_5/2_)	Sn^(Pt)^ (3d_5/2_)	Sn^(Pt)^ (3d_3/2_)	SnO_*x*_ (3d_5/2_)	SnO_*x*_ (3d_3/2_)
Bkg	Shirley	Shirley	Shirley	Shirley	Shirley	Shirley	Shirley	Shirley	Shirley
peak shape	GL (30)	LA (1.2,85,70)	LA (1.2,85,70)	GL (30)	GL (30)	GL (30)	GL (30)	LA (3,2,0)	LA (3,2,0)
area	3420	198	149	711	533	2571	1714	23,989	15,993
fwhm	3.6	1.2	1.2	1.7	1.7	2.9	2.9	1.9	1.8
position	73.9	69.8	73.2	73.2	76.6	485.8	494.3	486.2	494.7

SnPt_S-Macro (f)							
	Al 2p	Pt^0^ (4f_7/2_)	Pt^0^ (4f_5/2_)	PtO_2_ (4f_7/2_)	PtO_2_ (4f_5/2_)	SnO_*x*_ (3d_5/2_)	SnO_*x*_ (3d_3/2_)
Bkg	Shirley	Shirley	Shirley	Shirley	Shirley	Shirley	Shirley
peak shape	GL (30)	LA (1.2,85,70)	LA (1.2,85,70)	GL (30)	GL (30)	LA (3,2,0)	LA (3,2,0)
area	769	310	233	237	177	1732	1154
fwhm	2.1	3.5	3.3	1.9	1.9	2.3	2.3
position	73.7	69.7	73.0	75.0	78.4	486.8	495.2

SnPt_S-Macro (a)					
	Al 2p	Pt^0^ (4f_7/2_)	Pt^0^ (4f_5/2_)	SnO_*x*_ (3d_5/2_)	SnO_*x*_ (3d_3/2_)
Bkg	Shirley	Shirley	Shirley	Shirley	Shirley
peak shape	GL (30)	LA (1.2,85,70)	LA (1.2,85,70)	LA (3,2,0)	LA (3,2,0)
area	675	821	616	7387	5051
fwhm	2.0	2.2	2.2	2.7	2.7
position	73.6	70.3	73.6	486.6	495.3

SnPt_S-Macro (s)							
	Al 2p	Pt^0^ (4f_7/2_)	Pt^0^ (4f_5/2_)	PtO_2_ (4f_7/2_)	PtO_2_ (4f_5/2_)	SnO_*x*_ (3d_5/2_)	SnO_*x*_ (3d_3/2_)
Bkg	Shirley	Shirley	Shirley	Shirley	Shirley	Shirley	Shirley
peak shape	GL (30)	GL (30)	GL (30)	GL (30)	GL (30)	LA (3,2,0)	LA (3,2,0)
area	543	773	580	437	328	5770	3847
fwhm	1.7	2.6	2.6	2.7	2.7	2.9	2.9
position	73.6	70.4	73.7	75.1	78.4	486.2	494.7

The analysis of the Pt 4f peaks of the 10B20S70A-supported catalyst (Figure 7, left) shows that the fresh sample contains surface Pt (71.6%) and PtO_2_ (28.4%), while in the activated sample, only Pt^0^ was observed, confirming the complete reduction of Pt. During reaction, a partial oxidation of Pt^0^ to PtO was produced, according to the results obtained for the spent sample (19.8% Pt, 80.2% PtO). The binding energy of metallic Pt was lower than theoretical value (71 eV), which may indicate the formation of Pt–Sn alloy phases, as the differences in electronegativity of Pt and Sn could lead to charge transfer from the less-electronegative Sn to the more-electronegative Pt.^40,41^ The Sn core-level spectra (Figure 7, right) exhibit one symmetrical band at 486 eV that can be ascribed to Sn in a Pt environment (Sn^(Pt)^).^42^ The positive core-level shift of the Sn^(Pt)^ feature is consistent with electron exchange between the Pt and Sn atoms in an alloy environment.^43,44^ In the spent SnPt_10B20S70A, a broadening of the peak was detected because of the partial segregation of tin to the surface from the Pt–Sn alloy.^45,46^ In addition, in SnPt_10B20S70A samples, an extra peak, related to the Auger Na KLL, was detected, being more important in the activated and spent samples. This Na came from some contamination of the sample that migrated to the surface when the catalyst was activated.

The same platinum and tin species were observed in fresh SnPt_S-Macro (Pt, PtO_2_, and SnO_*x*_), where just sepiolite was used as the support. Pt content was 46.5%, while PtO_2_ represented 53.5%. Similarly, the activation process completely reduced the platinum. In the used sample, some of the Pt was oxidized to PtO_2_ (63% Pt, 27% PtO_2_).

Considering the fitting of the SnPt_10B20S70A fresh sample, a small peak was detected at 70.5 eV, which has to be related to Pt^0^ (4f_7/2_). Consequently, a contribution of Pt^0^ 4f_5/2_ has to be present also. Both peaks were fitted considering a LA(1.2, 85,70) peak (https://www.xpsfitting.com/search/label/Platinum), a distance between the peaks of 3.35 eV and a 2:3 area ratio. There is also a small peak around 78 eV that has to be related to PtO_2_ (4f_5/2_) so the corresponding 4f_7/2_ contribution has to be present. Finally, for the fitting of this complex spectra, we have to consider also the contribution of Al 2p which is present in the support.

Regarding the activated sample, a shoulder around 70.8 eV was detected that can be related to Pt^0^ (4f_7/2_). Then, the corresponding 4f_5/2_ contribution also has to be present. By considering a third peak (Al 2p), the fitting matched with the final shape with the spectra. Again, the 2:3 area ratio and the 3.35 eV distance between the doublet was considered, but this time, the shape of the peaks was GL(30), following xps fitting recommendations (https://www.xpsfitting.com/search/label/Platinum).

The spent sample also exhibited the Pt^0^ (4f_7/2_) contribution and then the 4f_5/2_ part. Considering only a third peak related to Al 2p, it is not possible to obtain the shape of the spectra, so other contributions have to be considered. No peak or shoulder ascribed to PtO_2_ was detected so the two final contributions have to be related to PtO.

Table S4 summarizes the surface chemical analysis for all the samples, with the Pt/Sn molar ratio being lower than the bulk ratio (0.43) in almost all the cases. This indicates an enrichment of Sn in the surface. However, in fresh SnPt_S-Macro, the ratio is similar to the bulk. In the fresh and the activated samples, the Pt/Sn molar ratios were similar, but it increased after reaction, which implies an increase of the Pt surface concentration.

**Figure 9:**
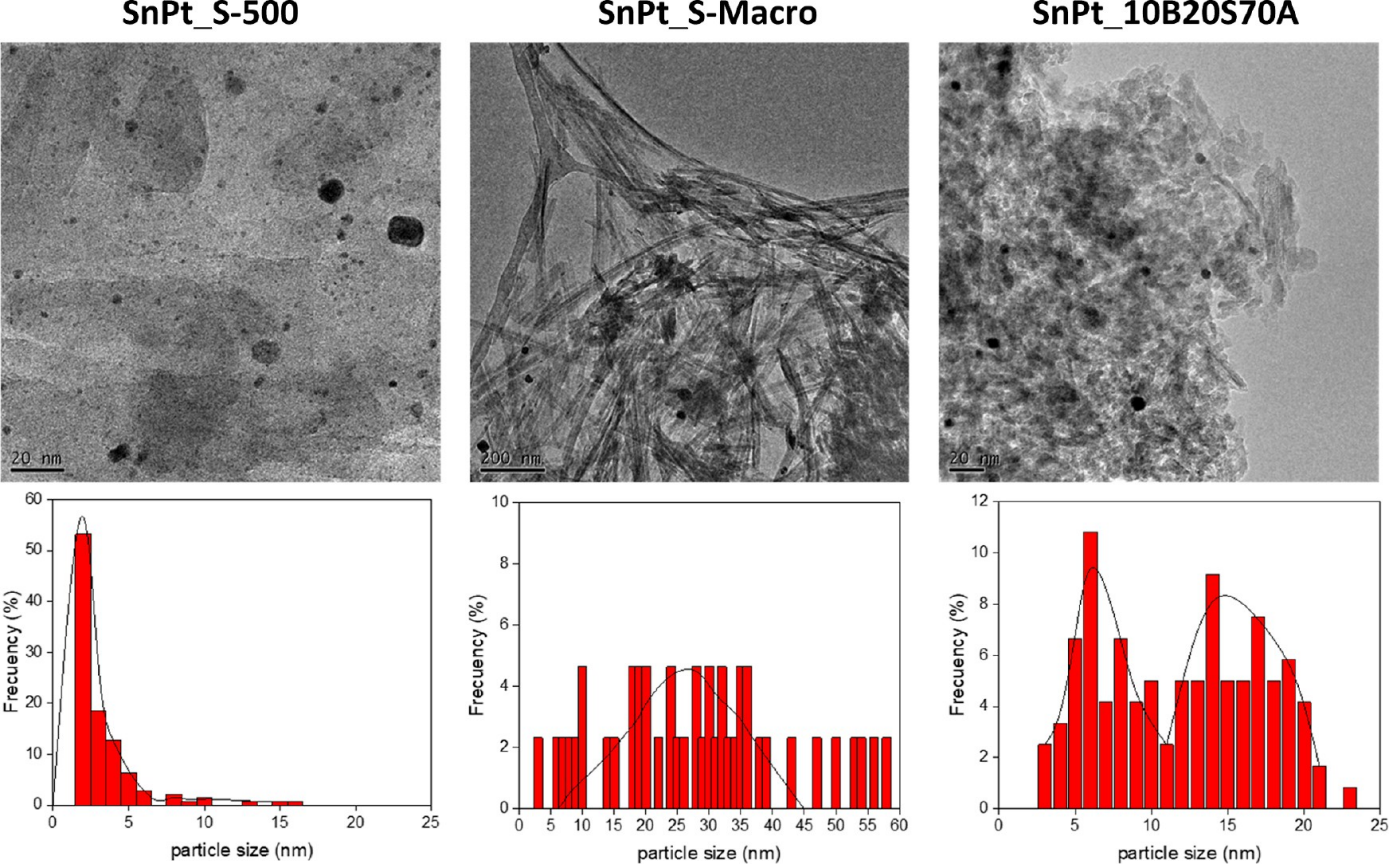
TEM images (top) and particle size distribution (bottom) of fresh SnPt_S-500, SnPt_S-Macro, and SnPt_10B20S70A.

TEM images of fresh SnPt_S-500, SnPt_S-Macro, and SnPt_10B20S70A are displayed in Figure 9 together with the particle size distribution. The narrowest particle size distribution and the smaller particles were observed in the sample SnPt_S-500 (average size = 2 nm), while SnPt_S-Macro exhibits the broadest distribution and the biggest particles (average size = 27 nm). SnPt_10B20S70A has a bimodal distribution with average sizes of 6 and 16 nm.

**Figure 10:**
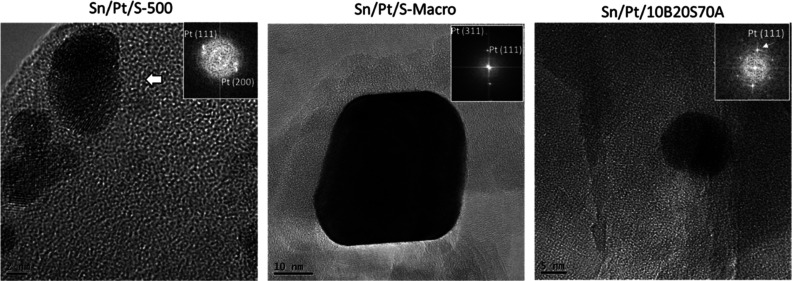
TEM images and FFT pattern (inset) of fresh SnPt_S-500, SnPt_S-Macro, and SnPt_10B20S70A.

More details were derived from fast Fourier transform (FFT) analysis performed in high resolution TEM (Figure 10, the inset shows the corresponding FFT pattern) used to calculate the lattice distance in the crystals. It was confirmed that Pt was mostly present as Pt^0^ in the fresh catalysts.

**Figure 11:**
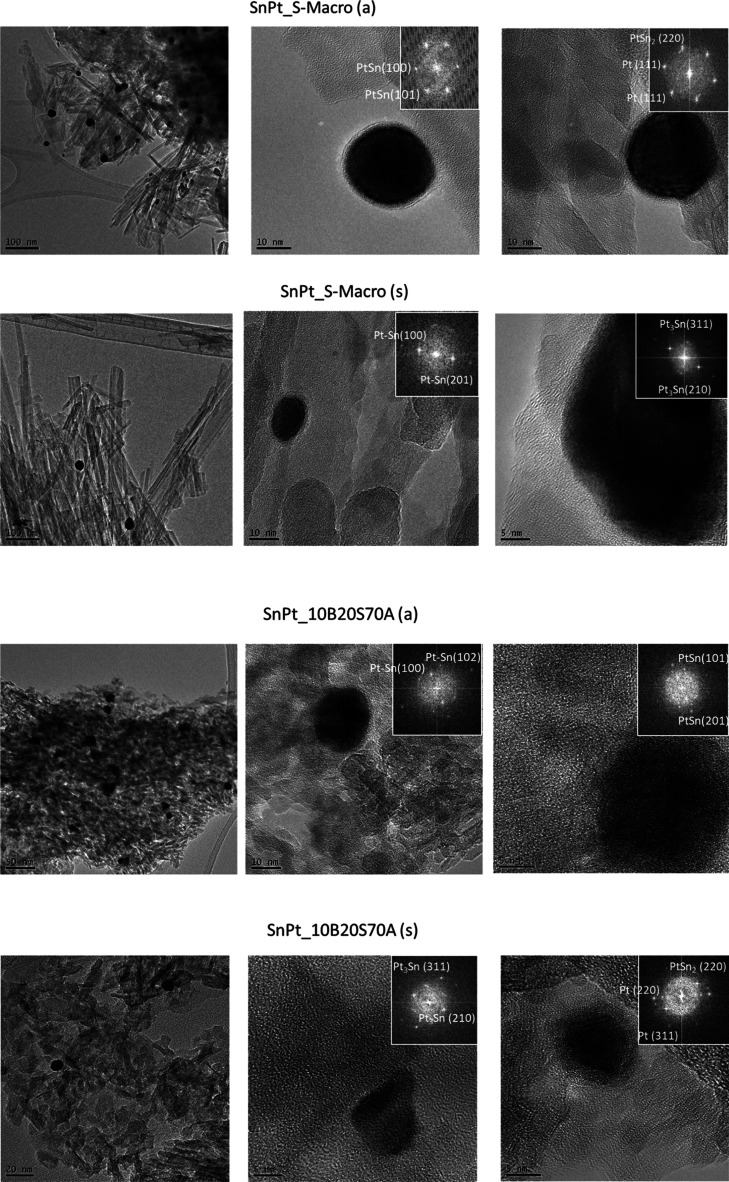
TEM images and FFT pattern (inset) of reduced and used SnPt_S-Macro and SnPt_10B20S70A.

The reduced and spent samples of SnPt_S-Macro and SnPt_10B20S70A, with better catalytic performance, were also characterized by HR-TEM (Figure 11), and the formation of several Pt–Sn alloys was observed, in agreement with XPS and XRD data. In the reduced SnPt_S-Macro sample, the lattice distance coincides with PtSn [0.3 nm related to the (101) plane] and PtSn_2_ [0.23 nm related to the (220) plane] alloys. The spent SnPt_S-Macro, used as a catalyst for PDH for 24 h, showed Pt–Sn alloys, with lattice distances ascribed to PtSn [0.36 and 0.17 nm for the (100) and (201) planes, respectively] and Pt_3_Sn [0.12 and 0.23 nm for the (311) and (210) planes]. Differently, in the case of the reduced SnPt_10B20S70A catalyst, only the PtSn alloy was detected, while after reaction, Pt nanoparticles were observed together with PtSn_2_ and Pt_3_Sn alloys.

**Figure 12:**
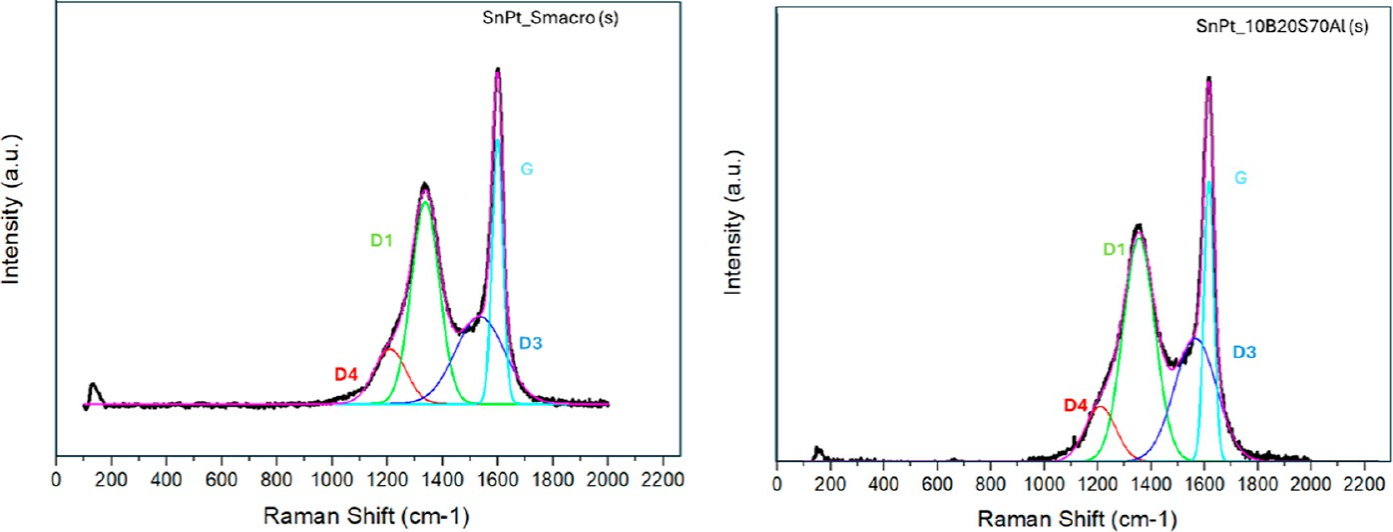
Raman spectra (black) of SnPt_S-Macro (s), on the left, and SnPt_10B20S70A (s), on the right. Fitting (magenta) and identification of the G (light blue), D1 (green), D3 (dark blue), and D4 (red) bands related to coke.

Finally, the spent catalysts from the stability tests were characterized by TGA analysis and Raman spectroscopy to evaluate the coke deposits formed on the surface of the catalyst during the reaction. In the Raman spectra of both SnPt_S-Macro and SnPt_10B20S70A spent catalysts (Figure 12), four main bands can be identified: the G band around 1600 cm^–1^ attributed to highly crystalline carbonaceous material (i.e., a graphitic lattice), the D1 band around 1360 cm^–1^ attributed to in-plane defects and heteroatoms in the carbon lattice, the D3 band around 1500 cm^–1^ attributed to amorphous carbon (out-plane defect), and the D4 band around 1210 cm^–1^ attributed to disordered graphitic lattice.^47^

**Figure 13:**
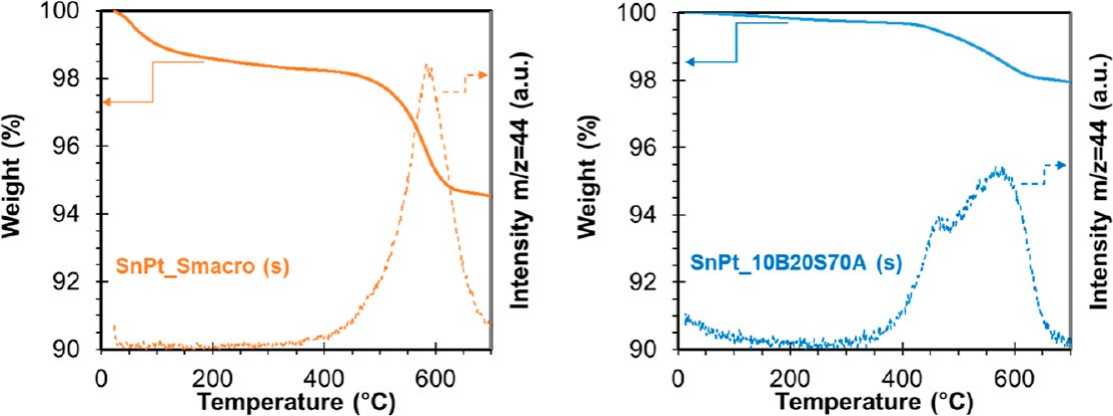
TG-MS profile (*m*/*z* = 44) of spent SnPt_10B20S70A and SnPt_S-Macro catalysts from 20 to 700 °C in air flow (100 N mL min^–1^).

The coke graphitization degree, calculated as the ratio between the area under the G band and the sum of the areas under the A_D1_ and A_D4_ bands, was 0.56 and 0.80 for SnPt_10B20S70A and SnPt_S-Macro, respectively. The lower graphitization degree of the catalyst with alumina in the support corresponds to a less ordered structure of the carbonaceous species deposits, suggesting that they might be removed by oxidation at lower temperatures. These have been verified by thermogravimetry under air flow. In Figure 13, the TGA-MS profile of SnPt_10B20S70A_spent and SnPt_S-Macro_spent catalysts shows a weight loss with an onset temperature of 330 °C attributed to coke combustion that corresponded to 1.7 and 3% of the spent sample weight of SnPt_10B20S70A (Figure 13a) and SnPt_S-Macro (Figure 13b), respectively. The CO_2_ profile (*m*/*z* 44) is different for the two spent catalysts: in the case of SnPt_10B20S70A, two types of carbon residue can be found, centered at 465 and 585 °C, whereas SnPt_S-Macro showed only one peak centered at 600 °C. As it is well-known, the relative position of TPO peaks is highly associated with the intrinsic properties and location of coke deposits. The peak around 600 °C can be attributed to graphite-like carbon, which blocks the active sites more easily than aromatic carbon, which only appears in SnPt_10B20S70A (465 °C). The presence of aromatic carbon resulting from the polymerization of alkane/alkene is observed on the metallic site.^48^

**Figure 14:**
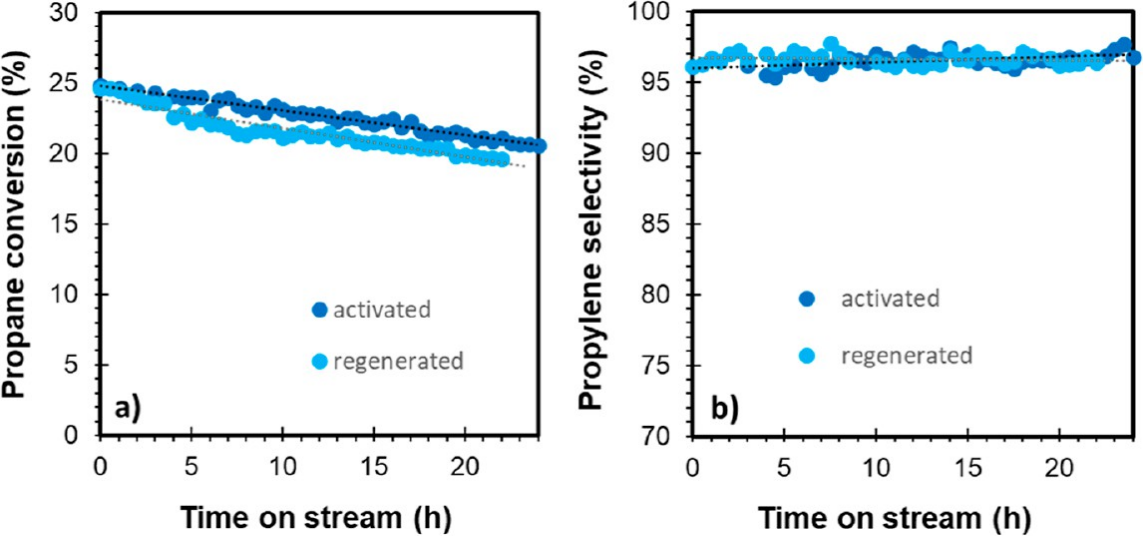
Stability/regeneration/stability cycle. Propane conversion (a) and propylene selectivity (b) vs time. SnPt_10B20S70A catalyst (*T* = 550 °C, *P* = 1 bar, WHSV = 4 h^–1^). Regeneration performed at 500 °C with increasing O_2_ concentration.

This very interesting result further evidences the good performance of the SnPt_10B20S70A catalyst, which is able not only to reduce the deactivation rate but also to form a more reactive coke. In this way, the catalyst can be regenerated at lower temperatures. A preliminary evaluation of the regenerability of this catalyst was performed in a stability/regeneration/stability cycle. Each of the two stability tests had a duration of 24 h under the same operating conditions of *T* = 550 °C, *P* = 1 bar, WHSV = 4 h^–1^. The results of propane conversion and propylene selectivity versus time reported in Figure 14 practically overlap, evidencing the full activity recovery after regeneration.

## 4 Discussion

This study consists of two complementary parts; first, we evaluate the effect of the addition of magnesium oxide to a mesoporous silica support for SnPt-based catalysts in the PDH reaction. Then we design a shaped SnPt-catalyst supported on a mixture of silica, alumina, and magnesium oxide that is adequate for industrial application. Alumina and silica are among the most used supports for PDH catalysts; in particular, systems based on mixed magnesium and aluminum oxide have shown high stability and good selectivity to propylene,^49^ whereas mesoporous silica-based supports have several advantages in terms of thermostability and availability,^9^ and some natural mesoporous magnesium silicates are excellent and cheap binders for extrusion of shaped bodies.^50^ Our results indicate that the combination of silica with magnesium and aluminum oxide may be useful for the design of industrial PDH catalysts. Figure 1, left, demonstrates for the selected Sn–Pt loading that the addition of magnesium oxide to the mesoporous silica support improves the performance of catalysts in terms of both conversion and selectivity to propylene. Although the addition of a base such as magnesium oxide is known to improve the performance of catalysts in terms of stability and resistance to coke formation,^27^ the effect on activity is less obvious. From the characterization of the catalysts, it is evident that the impregnation of magnesium oxide causes a decrease in the specific surface area, probably by partially blocking the silica support mesopores, making them less accessible. Certainly, a decrease in the specific surface area cannot be beneficial for catalysis purposes. Much more interesting is the comparison among the CO_2_-TPD desorption profiles of the SnPt_SiO_2_, SnPt_3MgSiO_2_, and SnPt_7MgSiO_2_ catalysts: not only the amount of basic sites increases with the Mg content (see Table S3), but also the CO_2_ desorption peaks of the Mg-containing catalysts are located at lower temperature (see Figure 6a), corresponding to weaker Brønsted basic sites. The trend of the amount of weak Brønsted basic sites, SnPt_SiO_2_ < SnPt_3MgSiO_2_ < SnPt_7MgSiO_2_, is reflected in the trend of catalyst activity.

Encouraged by these results, attention was focused on the preparation of efficient shaped catalysts by using supports in pellet form that contain magnesium and silicon oxide from natural clays, such as sepiolite (S-500 and S-Macro supports). Moreover, a special formulation containing sepiolite, bentonite, and alumina (10B20S70A support^29^) was also tested. All of the shaped catalysts have shown good results in terms of both propane conversion and propylene selectivity, with a very similar selectivity to coke (Figure 1, right). As in the case of the mSiO_2_-based catalysts, the catalytic activity seems related to the basic sites amount (see Figure 6b and Table S3). Both SnPt_S-Macro and SnPt_10B20S70A show propane conversion values very close to the thermodynamic equilibrium, although a long-term stability experiment (Figure 2) evidenced a slightly slower deactivation of the latter. Contrary to what can be expected, the lowest activity was detected over SnPt_S500 catalysts, which was the catalyst with the lowest Pt particle size (2 nm). This fact may be explained by the presence of strong basic sites that may hinder propane adsorption during the dehydrogenation reaction as suggested by other authors.^39^ On the SnPt_10B20S70A catalyst exhibited the highest catalytic activity and stability, probably due to its higher amount of total basic sites, but with a lower strength. The presence of two particle size domains (6 and 16 nm) leads to good activity due to the presence of very small particles, but a slightly lower selectivity due to broader particles that favor side reactions such as hydrogenolysis and coking. Moreover, the phase transition of Pt–Sn alloy also plays an important role in affecting catalytic performance: the stability of the supported Pt–Sn catalyst depends on the type of Pt–Sn alloy, where the PtSn phase is more effective than the Pt_3_Sn phase for PDH.^15^ In addition, a core–shell structure that originated from phase segregation was clearly observed on SnPt_S-Macro. This encapsulation inhibited the dehydrogenation activity resulting in gradual catalyst deactivation, as previously suggested by other authors.^35^

Raman and TG-MS analyses of the spent catalysts highlighted the formation of coke in both samples (Figures 12 and 13) but in lower amounts and with a lower coke graphitization degree for SnPt_10B20S70A, than for SnPt_S-Macro, corresponding to a less ordered structure of the carbonaceous species deposits that are thus oxidized at lower temperatures. In the regeneration tests of the spent catalysts in ambient oxidant, a maximum of CO_2_ formation appears around 600 °C (Figure 13); however, the SnPt_10B20S70A catalyst, differently from the SnPt_S-Macro one, has shown the formation of an additional peak at 465 °C, a sign of the formation of aromatic carbon that burnt under milder temperature conditions, avoiding stressing the catalysts during the regeneration step. In a preliminary stability/regeneration/stability test, the spent SnPt_10B20S70A catalyst recovered its activity after regeneration, both in terms of propane conversion and propylene selectivity. This last result is fundamental in view of an industrial application of the proposed catalyst. A comparison between the best catalyst of the present study (SnPt_10B20S70A) and some other Pt-based catalysts in the literature is reported in Table 3. The data evidenced the very good results of the developed catalyst, both in terms of propane conversion and propylene selectivity, even under different operating conditions and with a stream rich in propane.

**Table 3:** Comparison of the Catalytic Activity in PDH Reaction of Different Pt-Based Catalysts

catalyst	Pt wt %	*T* °C	WHSV h^–1^	components vol. %	propane conversion/%	propylene selectivity/%	ref
Pt–Sn/ZnAl_2_O_4_	3	600	32.4	C_3_H_8_ = 23, N_2_ = 77	22.0	98.0	(51)
Pt/TiO_2_–Al_2_O_3_	1	600	10	C_3_H_8_ = 26, H_2_ = 26, N_2_ = 48	25.9	89.5	(52)
10Zn_0.1_Pt/HZSM-5	0.1	525	0.24	C_3_H_8_ = 5, N_2_ = 95	43.0	93.0	(53)
Pt_3_Ga/CeO_2_–Al_2_O_3_	1	600	10	C_3_H_8_ = 26, H_2_ = 26, N_2_ = 48	32.2	99.6	(54)
Ga^δ+^Pt^0^/SiO_2_	4.37	550	98.3	C_3_H_8_ = 20, Ar = 80	31.9	99.0	(55)
PtSnNaCe/ZSM-5	0.5	590	3.0	C_3_H_8_ = 80, H_2_ = 20	36.0	92.6	(56)
In–Pt/Sn-SBA-15	1	580	4.05	C_3_H_8_ = 20, Ar = 80	46.9	99.0	(57)
Pt–Sn/B–ZrO_2_-10	0.35	550	3	C_3_H_8_ = 10, H_2_ = 10, N_2_ = 80	36.0	99.5	(58)
Pt–Ir/Mg(Al)O	1.91	600	51.9	C_3_H_8_ = 20, H_2_ = 20	17.5	88.7	(59)
Pt–Sn/CeO_2_	1	680	2.2	C_3_H_8_ = 16.7, H_2_ = 83.3	39.5	78.0	(60)
SnPt_10B20S70A	0.5	600	4	C_3_H_8_ = 80, H_2_O = 20	40	94	This work

## 5 Conclusions

This research contributes to the development of highly efficient and stable industrially relevant catalysts for propene dehydrogenation. It underscores the importance of the supporting material and explores various strategies to obtain porous supports with adequate basicity at a low cost to enhance catalytic performance, ultimately addressing the challenges associated with propylene production in the chemical industry.

In this work, we have shown how the acid–base characteristics of silica-based supports can be adjusted by Mg promotion, thus obtaining better performances both in terms of propane conversion and selectivity to propylene. Moreover, the basicity of the support affects coke formation, which in turn influences long-term catalytic performance. Mixed supports based on sepiolite, bentonite, and alumina can guarantee high resistance to the coke formation; furthermore, the relatively low temperature required for regeneration, due to the formation of carbon with lower combustion temperature, reduces the stress of the catalysts during the regeneration phase, improving their duration. The samples SnPt_S-Macro and SnPt_10B20S70A achieved propane conversions of 23 and 24%, respectively. The former showed a higher deactivation rate, with a final propane conversion of about 16% after 24 h, while the latter catalyst showed a propane conversion of about 21% after 24 h of TOS.

## Supporting Information Available

It contains The Supporting Information is available free of charge at https://pubs.acs.org/doi/10.1021/acs.iecr.3c04089.

Textural properties, chemical composition, CO_2_-TPD results, XPS fitting details, surface composition, EDS results, XRD patterns, gas phase chromatogram, and TPO of spent SnPt_10B20S70A catalyst (PDF)
