# Supplementary material for: Support Screening to Shape Propane Dehydrogenation SnPt-Based Catalysts
Source: Ind Eng Chem Res. 2024 Sep 12;63(38):16269–84. doi: 10.1021/acs.iecr.3c04089 (PMC11440506; doi:10.1021/acs.iecr.3c04089)
Supplement: Supplementary file 1 — ie3c04089_si_001.pdf [file ie3c04089_si_001.pdf]

Supporting Information

Support screening to shape propane dehydrogenation SnPt-based catalysts

Giovanni Festa[1], Ana Serrano-Lotina[2], Eugenio Meloni*[1], Raquel Portela*[2], Concetta Ruocco[1], Marco Martino[1], Vincenzo Palma[1]

[1] University of Salerno, Department of Industrial Engineering, Via Giovanni Paolo II 132, 84084 Fisciano, SA, Italy.

[2] Instituto de Catalisis y Petroleoquimica (ICP), CSIC, C/ Marie Curie 2. 28049 Madrid, Spain.

* Correspondence: emeloni@unisa.it; raquel.portela@csic.es

Content:

1. **Tables S1 to S5:**

- Table S1. Textural properties of the calcined supports
- Table S2. Chemical and textural properties of the calcined catalysts
- Table S3. CO_2 uptake distribution over the SnPt_based catalysts in CO_2 temperature-programmed desorption experiments
- Table S4. Surface composition of fresh, activated and spent SnPt_10B20S70A and SnPt_ S-Macro samples, evaluated by XPS

2. **Figures S1 to S4**

- Figure S1. EDS of SnPt_10B20S70A (top) and SnPt_S-Macro (bottom), fresh (left) and spent (right).
- Figure S2. XRD pattern of metallic Pt (JPDS # 00-004-0802), PtO (JPDS # 00-042-0866), PtO_2 (JPDS # 00-023-1306), Pt_3Sn (JPDS # 00-035-1360), PtSn (JPDS # 00-025-0614), $PtSn_2$ (JPDS # 00-007-0371), SnO_2 (JPDS # 00-029-1484), SnO (JPDS # 00-006-0395), and Sn0 (JPDS # 00-004-0673) .
- Figure S3. Gas phase chromatograph obtained during a PHD test showing the absence of gas-phase by-products
- Figure S4. Controlled coke oxidation test performed on the spent SnPt_10B20S70A catalyst

1 Tables:

Table S1. Textural properties of the calcined supports

Support	S_{BET}[1] (m^2g^{-1})	Pore V[2] (mL g^{-1})
$mSiO_2$	546	0.31
3Mg_$mSiO_2$	413	0.26
7Mg_$mSiO_2$	268	0.23
S-500	164	0.29
S-Macro	141	0.83
10B20S70A	165	0.63

[1]Evaluated with nitrogen adsorption-desorption isotherms at 77 K; [2] evaluated with mercury intrusion porosimetry.

Table S2. Chemical and textural properties of the calcined catalysts

Catalyst	S_{BET} [1] (m^2g^{-1})	Pore V[2] (mL g^{-1})	Pt loading[3] (wt %)	Sn loading[3] (wt %)
SnPt_SiO_2	531	0.28		
SnPt_3MgSiO_2	355	0.23	0.54	0.75
Sn-Pt-7MgSiO_2	268	0.19	0.53	0.72
SnPt_S-500 (f)	92	0.25	0.53	0.70
SnPt_S-Macro (f)	126	0.73	0.53	0.75
SnPt_10B20S70A (f)	162	0.43	0.54	0.77
SnPt_S-Macro (s)	110	0.73		
SnPt_10B20S70A (s)	147	0.41		

[1]Evaluated with nitrogen adsorption-desorption isotherms at 77 K; [2] evaluated with mercury intrusion porosimetry. [3] evaluated with XRF

Table S3. CO_2 uptake distribution over the SnPt_based catalysts in CO_2 temperature-programmed desorption experiments

Sample	Desorbed CO_2 (μmol·$g_{catalyst}^{-1}$)		
	**100 < T < 250°C** (weak basic sites)	**250 < T < 400 °C** (medium-strength basic sites)	**400 < T < 600 °C** (strong basic sites)
SnPt_SiO_2	0.66 (T = 140 °C) + 0.52 (T = 245 °C)		
SnPt_3MgSiO_2	2.95 (T = 116°C)	1.49 (T = 321 °C)	
SnPt_7MgSiO_2	1.24 (T = 92 °C) + 2.99 (T = 128 °C)		0.69 (T = 519 °C)
SnPt_S-Macro	0.99 (T = 100 °C) + 2.74 (T = 145 °C)		
SnPt_S-500	0.88 (T = 136 °C) + 0.47 (T = 211 °C)		3.24 (T = 538 °C)
SnPt_10B20S70A	13.3 (T = 144 °C) + 15.4 (T = 189 °C)		2.43 (T = 513 °C)

Table S4. Surface composition of fresh, activated and spent SnPt_10B20S70A and SnPt_ S-Macro samples, evaluated by XPS

Catalyst	Pt loading[3] (wt %)	Sn loading[3] (wt %)	Pt/Sn atomic ratio
SnPt_10B20S70A (f)	0.43	2.00	0.13
SnPt_10B20S70A (a)	0.14	0.74	0.12
SnPt_10B20S70A (s)	0.25	0.61	0.25
SnPt_ S-Macro (f)	0.10	0.16	0.40
SnPt_S-Macro (a)	0.16	0.71	0.14
SnPt_ S-Macro (s)	0.23	0.53	0.26

2 Figures:

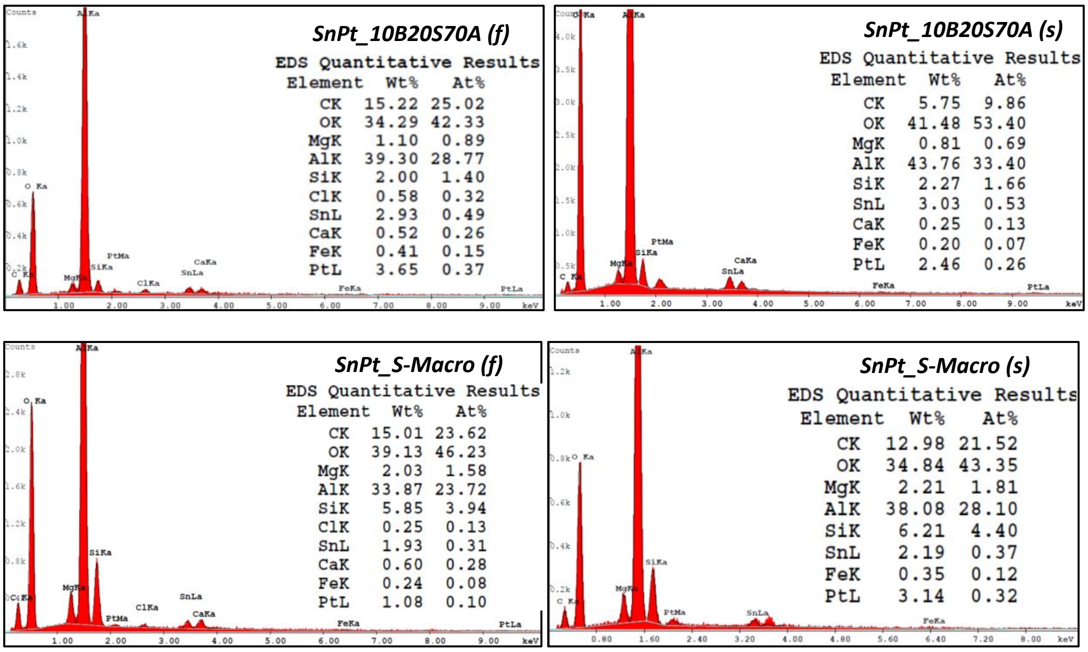

Figure S1. EDS of SnPt_10B20S70A (top) and SnPt_S-Macro (bottom), fresh (left) and spent (right).

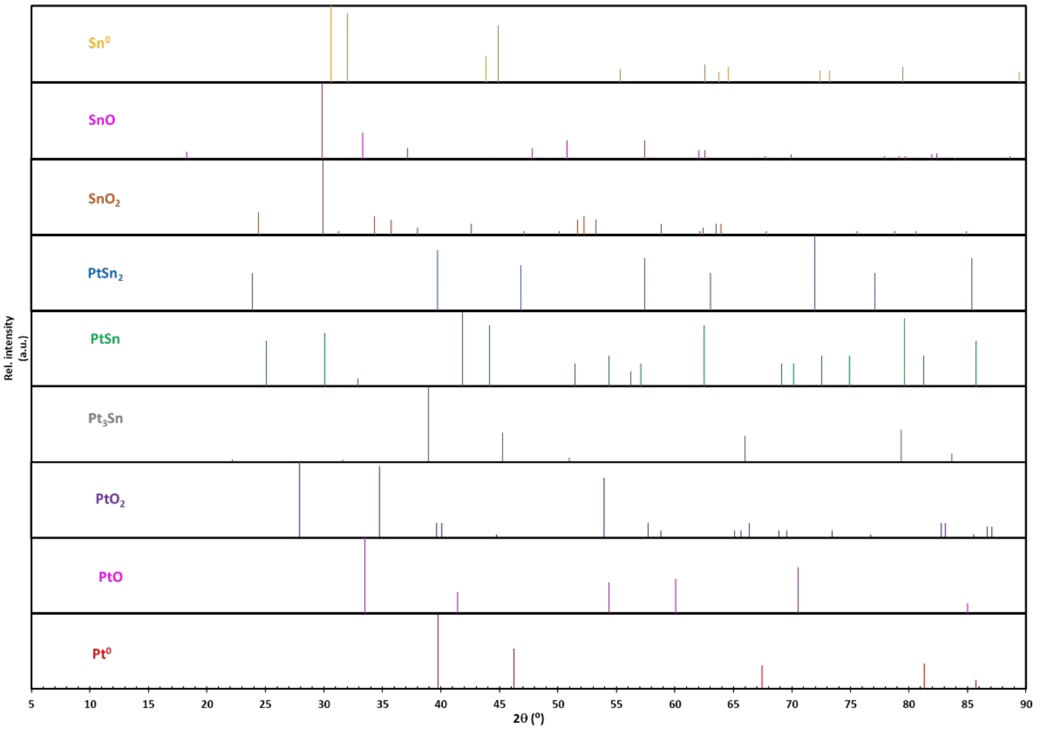

Figure S2. XRD pattern of metallic Pt (JPDS # 00-004-0802), PtO (JPDS # 00-042-0866), PtO_2 (JPDS # 00-023-1306), Pt_3Sn (JPDS # 00-035-1360), PtSn (JPDS # 00-025-0614), $PtSn_2$ (JPDS # 00-007-0371), SnO_2 (JPDS # 00-029-1484), SnO (JPDS # 00-006-0395), and Sn^0 (JPDS # 00-004-0673) .

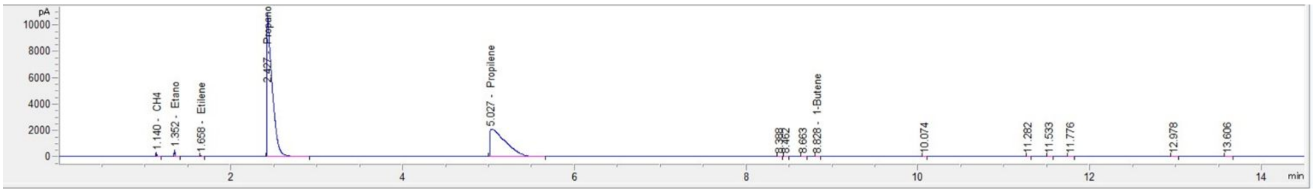

Figure S3. Gas phase chromatograph obtained during a PHD test showing the absence of gas-phase by-products.

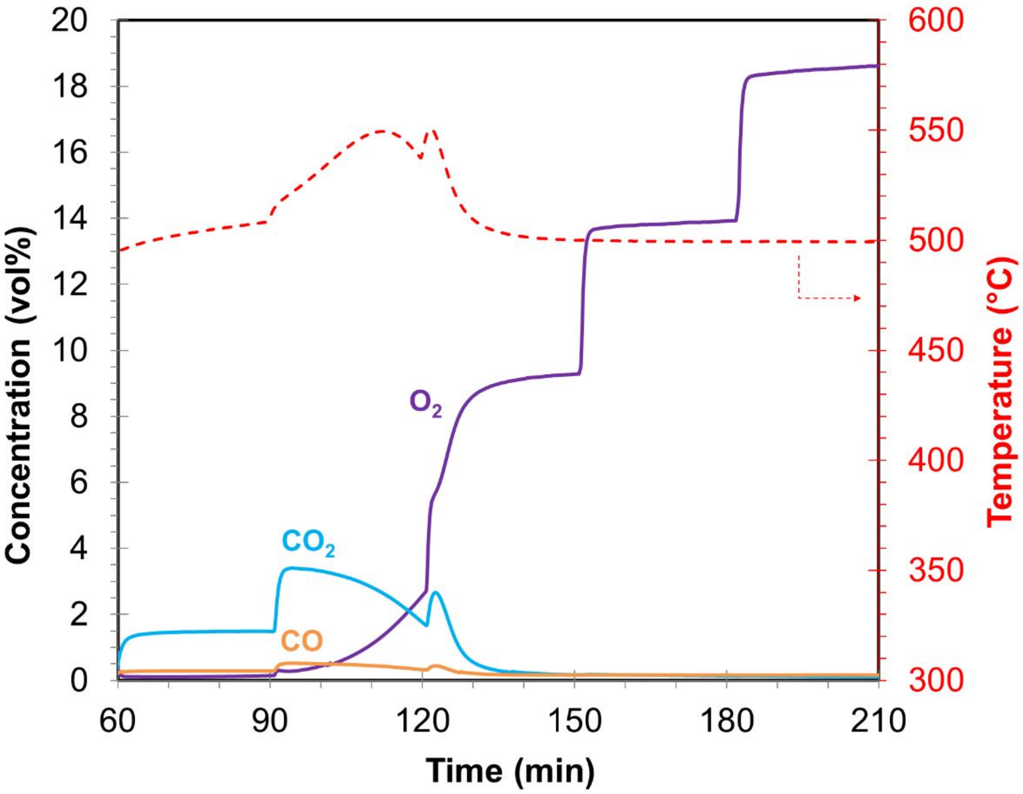

Figure S4. Controlled coke oxidation test performed on the spent SnPt_10B20S70A catalyst.
